# Mapping gender stereotypes: a network analysis approach

**DOI:** 10.3389/fpsyg.2023.1193866

**Published:** 2023-07-18

**Authors:** Ángel Sánchez-Rodríguez, Eva Moreno-Bella, Efraín García-Sánchez

**Affiliations:** ^1^Department of Social Psychology and Anthropology, University of Salamanca, Salamanca, Spain; ^2^Department of Social and Organizational Psychology, National University of Distance Education, Madrid, Spain; ^3^Department of Social Psychology, University of Granada, Granada, Spain

**Keywords:** gender stereotypes, gender metastereotypes, in-group stereotypes, networks approach, social perception

## Abstract

**Introduction:**

Stereotypes have traditionally been considered as “mental pictures” of a particular social group. The current research aims to draw the structure of gender stereotypes and metastereotype schemes as complex systems of stereotypical features. Therefore, we analyze gender stereotypes as networks of interconnected characteristics.

**Method:**

Through an online survey (*N* = 750), participants listed the common female and male features to build the structure of the gender stereotypes. Participants also listed the common features of how members of one gender think they are viewed by people of the other gender to build the structure of gender metastereotypes.

**Results:**

Our results suggest that female stereotypes are characterized by a single community of features consistently associated such as *intelligent, strong*, and *hardworkers*. Female metastereotype, however, combines the previous community with another characterized by *weak* and *sensitive*. On the contrary, the male stereotype projected by women is characterized by a community of features associated such as *intelligent, strong*, and *hardworker*, but male in-group stereotypes and metastereotypes projected by men are a combination of this community with another one characterized by features associated such as *strong, chauvinist*, and *aggressive*.

**Discussion:**

A network approach to studying stereotypes provided insights into the meaning of certain traits when considered in combination with different traits. (e.g., strong-intelligent vs. strong-aggressive). Thus, focusing on central nodes can be critical to understanding and changing the structure of gender stereotypes.

## 1. Introduction

The research about gender stereotypes has been a longstanding storyline in social science and has inspired much research in gender theories (Eagly, 1987; Wood and Eagly, 2015). Social psychologists have focused on studying gender stereotypes because they are a key construct in explaining the differences between women and men regarding their behavior and attitudes, as well as ideologies that perpetuate gender inequality (Eagly, 1987; Ellemers, 2018). Similarly, the stereotypes that people of one gender have about the way in which they are viewed by people of another gender—i.e., gender metastereotypes—are key to determine intergroup relations (Gómez, 2002; Babbitt et al., 2018). Therefore, understanding current gender stereotypes and metastereotypes influences how people evaluate others and themselves, which condition gender intra- and intergroup relationships (Ellemers, 2018).

Stereotypes have been traditionally considered “mental pictures” of a specific social group (Lippmann, 1922). Although initially the research was focused on finding the features attributed to gender, it was found that those features could be organized in dimensions (Broverman et al., 1972; Fiske et al., 2002). From that moment, the research on gender stereotypes has grown on the rationale that the content of stereotypes consists of a few dimensions that can summarize a wide range of stereotypical features. Although this approach has profoundly enriched the understanding of stereotypes, it loses the information provided by single features and their interactions, which, as a whole, determine a more complex structure of gender stereotypes. In the current research, we aim to deepen the structure of current gender stereotypes by depicting them as complex systems, that is, as an ensemble of many elements which are interacting in a particular way, resulting in a robust organization (Ladyman and Wiesner, 2020). To address them as complex systems, we take a network approach, considering stereotypical features as the basic elements, to explore how they interact with each other and draw the structure of gender stereotypes. We proposed a bottom-up strategy to build the structure of gender stereotypes from the free responses of the participants, given that this strategy allows us to reflect the spontaneous current social representation that individuals embrace (Moscovici, 1984).

### 1.1. Gender stereotypes

Stereotypes have been defined as overgeneralized, rigid, and exaggerated beliefs about the characteristics, attributes, and behaviors of members of certain groups (Cardwell, 1996). People stereotype others in an attempt to understand their social world (Ellemers, 2018). Gender stereotypes have been considered as people's shared beliefs about the traits of women and men (Sczesny et al., 2019). A common task in social psychology is identifying the content of gender stereotypes, given that traditional gender stereotypes maintain and reinforce gender inequalities (Ellemers, 2018). Following the literature, men are traditionally stereotyped as being more *agentic* than women, whereas women are stereotyped as being more *communal* than men (Fiske et al., 2002; Hentschel et al., 2019). Agency and communion are the fundamental dimensions of human orientation and social judgment (Abele and Wojciszke, 2014). Agency refers to goal achievement and task functioning, emphasizing two facets: assertiveness and competence. Assertiveness reflects the motivational and purposive component of agency, whereas competence reflects the ability component. On the contrary, communion refers to the maintenance of relationships and the desire for affiliation, emphasizing the warmth and morality facets. Warmth refers to affective motives, and morality refers to benevolence, ethics, and social values (Abele and Wojciszke, 2014; Wojciszke and Abele, 2019). Similar differences in gender stereotypes have been proven across different cultures (Williams et al., 1999). Although it is common an emphasis on personality traits, there is also evidence of a multi-component construction of gender stereotypes. Deaux and Lewis (1983) identified components of gender stereotypes in traits, role behaviors, occupations, and physical appearance. Interestingly for the current research, Deaux and Lewis (1984) shown, in subsequent research, that those components dynamically implicate each other.

Stereotypes about social groups can be held by the in-group, which is defined as in-group stereotype. Whereas stereotyping out-groups is usually related to discrimination toward their members (Wilder, 1984), the in-group stereotype has implications for self-concept and self-categorization (Hogg and Turner, 1987). Therefore, stereotypes do not only influence how we evaluate others but also ourselves. Similarly, metastereotypes are conceptually distinct from in-group stereotypes because metastereotypes refer to individual group members' beliefs about how others view their group, whereas in-group stereotypes refer to individuals' beliefs about their own group (Gómez, 2002; Babbitt et al., 2018). A metastereotype is associated with feelings toward intergroup interaction and attitudes and assessments of the out-group members (Owuamalam et al., 2013).

Understanding the content of gender stereotypes requires recognizing the relevance of social roles (Eagly and Karau, 2002). According to the social role theory, gender stereotypes are built on social roles (Eagly, 1987). Traditionally, women and men have been segregated into different atmospheres: women in domestic work or care-related jobs, and men in leadership or skill-related jobs (Lippa et al., 2014). This unequal segregation enacts gender differences in the sense that people form their impressions of others through observing their behavior (Gilbert and Malone, 1995). Therefore, people create different images of women and men. In other words, everyday observations of the different roles of women and men underlie gender stereotypes (Eagly, 1987; Sczesny et al., 2019).

Because gender stereotypes are a result of people's beliefs and expectations of women and men in a given cultural context, they may change as the context changes (Diekman and Eagly, 2000; Sczesny et al., 2019). Given the advances toward gender equality and the decrease of the unequal distribution of women and men in different roles (Lippa et al., 2014; World Economic Forum, 2020), gender stereotypes are expected to reflect this change (Eagly and Karau, 2002). Some findings of stereotypical ascriptions for women and men have found that, indeed, there have been changes, but others that some stereotypes persist. For instance, Haines et al. (2016) tested whether gender stereotypes had changed over 30 years (1983–2014). Their findings suggested that people continued to rate women as more communal than men and rated men as more agentic than women, just as people did 30 years ago. Recent research in the Spanish context showed no evidence of stereotype changes in agentic and communal personality traits between 1985 and 2018 (Moya and Moya-Garófano, 2021). Similarly, Hentschel et al. (2019) found that gender stereotypes persisted in terms of communality features. However, they did not find differences by sex in independent and leadership roles, suggesting there have been changes in these components of the agency dimension. In this line of thinking, other results suggested an increase in perceived competence of women across time (Duehr and Bono, 2006; Eagly et al., 2020). The difference between assertiveness and competence within the agency dimension seems to be relevant nowadays because contemporary female stereotypes may reflect lower assertiveness than those of men but not competence (Sczesny et al., 2019). Similarly, women attributed to themselves less traditional feminine features in 2012 than in 1993; however, there was no significant difference in the features men attributed to themselves in the same period (Donnelly and Twenge, 2017).

Therefore, although some literature suggests that there have been changes in gender stereotypes, others suggest that they have remained stable for a long time. Despite changes in social roles that might work as a force for changing gender stereotypes, the nature of stereotypes also provides them with forces for remaining unchanged throughout time (e.g., through memory and attributional processes; Hilton and von Hippel, 1996). We should highlight that the evidence that gender stereotypes are changing, although slowly, suggests that they are dynamic systems. Here, we propose that in addition to dynamic systems, gender stereotypes can be understood as complex systems, which in turn can shed light on how they change (van Geert, 2019). Complex systems can be seen as an ensemble of elements that are interacting in a particular way, resulting in a robust organization (Ladyman and Wiesner, 2020). To understand the gender stereotype as complex systems, we use a network approach.

### 1.2. Stereotypes as a network of features

To understand how to work a complex system, we must understand not only how to work their elements but also how they act together as a whole (Bar-Yam, 1997). In the current research, we inspected the complex interaction of single features and how they draw the mental gender stereotype pictures in descriptive terms—i.e., descriptive gender stereotype. To do so, we drew the current research on the literature of the network approach. In psychology, the network approach posits indicators are not only passive items that reflect latent constructs but also they might form dynamic and complex systems because of the interactions among themselves (Cramer et al., 2010; Robinaugh et al., 2020). Therefore, from a network perspective, features that characterized gender stereotypes would not be interchangeable indicators that could be averaged. Instead, features of gender stereotypes take particular meanings depending on their position in the overall network and their interaction with other features (Cramer et al., 2010). Accordingly, we could shed light on the structure of the gender stereotypes and metastereotypes building the associative networks of features that reflect the gender schemes widespread in the society. We should note that this approach would be different from the construction of associative networks of the features in which are not assumed any underlying structure.

Gender stereotypes from a network approach would be built from a set of interacting features. It is likely that the network approach would reflect more accurately how stereotypes are represented in the mind as “mental pictures” (Sayans-Jiménez et al., 2019). Some initial research has already used network analyses to map stereotypes, but they are still based on their analysis of the general dimensions of the stereotypes' content (Grigoryev et al., 2019; Sayans-Jiménez et al., 2019), which limit the information provided by predefined categories. Therefore, in the current research, we modeled gender stereotypes using features as nodes instead of dimensions and based on people's free responses without constraining them to previous categories. We raised that understanding gender stereotypes as complex systems and addressing them from a network approach using features as their basic elements might shed light on several issues in the study of stereotypes.

First, gender stereotypes can be seen as complex systems (van Geert, 2019), which help us to understand their changes (Diekman and Eagly, 2000; Sczesny et al., 2019). For instance, in times of rapid social changes, as in current times, changes in gender stereotypes are unlikely to occur in all the people in the same way or at the same speed. Consequently, nowadays, traditional and modern forms of gender stereotypes might be coexisting in society. To address this point, we should explore the structure of gender stereotypes to test to what extent gender stereotypes are homogeneous or heterogeneous nowadays. Because the dimensional approach used to address this issue has limitations in accounting for the complexity of a wide variety of gender stereotypes, we can take a network approach to account for the coexistence of attributes linked to gender stereotypes. By considering gender stereotypes as the co-occurrence of several features people identify, we can explicitly model a network of attributes that depict people's gender perceptions. Similarly, network analyses provide tools to detect the network's underlying substructure, which is crucial in identifying clusters of attributes that are more likely to appear together—i.e., to check whether everyone raises a similar gender stereotype, which would suggest that they are homogeneous, or whether different groups in the population raise different gender stereotype, which would point out that gender stereotypes are heterogeneous.

Second, once we have built the structure of gender stereotypes, we can compare qualitatively the similarities and differences between those projected by women and men. For instance, female stereotypes can be projected by women—i.e., in-group stereotype—, by men, and by the beliefs that women have about the female stereotype projected by men—i.e., metastereotype. This distinction is important because the three forms of stereotypes might be related to different outcomes. Following the previous literature on stereotypes, the female in-group stereotype should be related to women's self-concept and self-categorization (Hogg and Turner, 1987); the female stereotypes held by men condition the relationship of men toward women (Wilder, 1984); and metastereotypes could influence women's attitudes toward men (Owuamalam et al., 2013). These three forms of stereotypes are likely to reinforce each other. What is unknown is to what extent they share a similar structure. We focus on the differences between gender stereotypes and metastereotypes held by male and female participants.

Third, a network perspective might help to qualify the meanings to attribute to each stereotypical feature according to co-occurring features. We know from Asch's (1946) classic studies that the impression formed toward someone when we defined him/her as intelligent, skillful, industrious, determined, practical, and cautious is quite different if we add *warm* or *cold* to the list. Indeed, the perception of a person would vary if people think she is intelligent and warm or intelligent and cold. This result suggests that features attributed to a person interact with each other to build their meanings rather than they are interpreted separately. Thus, the general impression that we build of others emerges from the interaction of the features that we attribute to them. Although this process happens from features to the interpretation (bottom-up), a parallel process could be happening from interpretation to attributed characteristics (top-down), which would be guided by schemes (e.g., gender schemes, Bem, 1981). We suggest that this process applies also to the groups when individuals attribute them stereotypical features, particularly to gender stereotypes. However, given that the mainstream approach did not take into account the interactions between features because considering them interchangeable indicators that could be averaged on general indices, we unknown how stereotypical gender features interact with each other and how representation is built. The network approach allows us to fill this gap using a co-occurrence matrix of features the participants mentioned to conduct the network analyses. This strategy allows us to account for the association of all the features simultaneously. In this way, we can check the interactions between features for a novel and more detailed understanding of gender stereotypes.

## 2. The present research

The current research aims to explore the structure of the current gender stereotypes from a network approach. Given that we are interested in the widespread schemes related to gender stereotypes and metastereotypes, we adopted a strategy that reflects the spontaneous current social representation that individuals embrace (Moscovici, 1984). Therefore, we used a free association technique to collect our data (Tsoukalas, 2006). Afterward, we conducted a bottom-up strategy to analyze our data applying network analyses to identify the pattern of associations among attributes, which describe the structure of gender stereotypes. Finally, we extended these analyses to gender metastereotypes to look at their structure and similarities with the in-group stereotype.

### 2.1. Methods

#### 2.1.1. Participants

We conducted an online study by sending an email to the university community of a city in southern Spain. There were 750 respondents to our study (512 women, 190 men, 5 others, and 43 missing gender information), who ranged from 18 to 64 years old (*M* = 24.85, *SD* = 7.78). There were 585 participants that indicated that Spanish was their native language (30 were not and 135 missing language information). There were no exclusions, so we include all the participants in the analyses. Participants' sociodemographic features are available in Supplementary material (Section S1).

#### 2.1.2. Procedure and measurement

All the procedures performed in this study were in accordance with the ethical standards of the institutional research committee. First, we asked participants about gender stereotypes of both men and women. We counterbalanced the order of presenting the gender stereotypes to avoid order effects. Given that we were interested in the descriptive gender stereotype, participants read the following instruction before providing their answers: “Think about all the women (men) you know—i.e., relatives, friends, work or university colleagues, women (men) who appear on television, on social networks, in books…—What are the features that all those women (man) have in common?” Participants were asked to write ten open-ended answers. We suggested answering for at least five features.

Afterward, we asked them about their gender, and according to their answer, we asked them about the metastereotype,^1^ that is, we were interested in how members from one gender think they are viewed by people from the other gender. Therefore, if they indicated they were women (men), we asked them to think about all the men (women) they knew. Then, participants were asked to write what those men (women) thought about women (men). They were to list these features in 10 open-ended responses. We encouraged them to answer for at least five features.

##### 2.1.2.1. Data processing

Given that participants provided open-ended answers, we obtained a high diversity of words referring to similar features. Thus, three researchers manually performed the lemmatization of the data corpus, which consisted in keeping the base form of each word (lemma) by removing the inflectional ending or word derivations participants used (e.g., friends, friendly, friendship, and friendliness indicate the same lemma, friend). This strategy is commonly used in linguistic processing to reduce the variability of words that capture the same meaning. Although this process might lead us to lose sight of some nuances, this is a practical procedure to simplify large amounts of information that can be redundant, which allows us to reach a more substantive interpretation. Furthermore, this process was performed manually, allowing researchers to detect potential ambiguities and select the most appropriate lemma according to the context.

Because our original data corpus was mostly composed of single words in Spanish, after the lemmatization, we automatically translated each word into English. Then, two researchers verified the translation's accuracy by reviewing whether those words fit the original meaning properly. This stage allowed a new check on the quality of lemmatization and appropriate use of words. The raw and coded material is available in the online Supplementary material (https://osf.io/cmf6a/).

**Figure 1:**
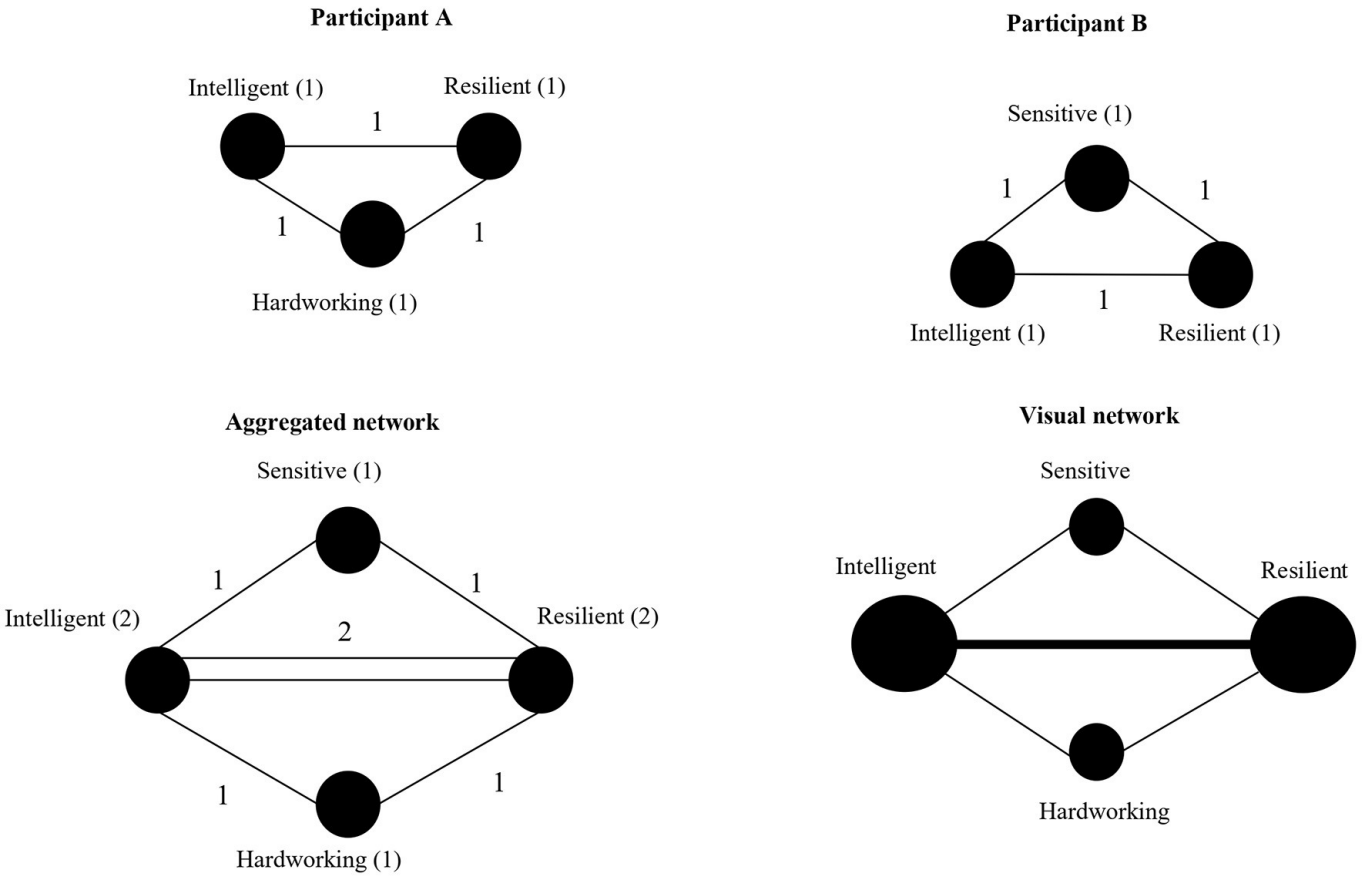
Example of co-occurrence network. Each circle represents one feature (node), and in parenthesis is the number of times that such attribute is mentioned (centrality degree); lines represent the edges, and the number next to the line represents the weight of each association. The upper figures indicate individual networks for two participants, and the bottom figure indicates the aggregated network per group.

We used the co-occurrence matrix of features the participants mentioned to conduct the network analyses. The network analysis strategy allowed us to account for the relationship of all the features simultaneously to reveal patterns of association. Networks are made up of two components: nodes and edges. Nodes correspond to participants' responses (i.e., features), and edges reflect the associations between nodes (i.e., features mentioned by the same person are connected). Thus, we had as many nodes as named features regardless of how many times these features were mentioned, and edges reflect the co-occurrence of every pair of features the same participant mentioned. To illustrate the procedure, imagine that a participant A answered three features of female stereotypes: intelligent, resilience, and hardworking (Figure 1, upper left). Therefore, we would have three nodes and three edges: intelligent-resilience, intelligent-hardworking, and resilience-hardworking. Another participant B answered three more features: intelligent, resilience, and sensitive (Figure 1, upper right). Therefore, the combination of those two personal networks provides an outcome graph composed of four nodes and five edges (Figure 1, bottom left). Finally, to visualize this network, the nodes' size increases according to the number of times they were mentioned, and edges are collapsed (Figure 1, bottom right). Note that the links between intelligent and resilience collapse, becoming one single edge with a weight equaling 2, and therefore, suggesting that this relationship is stronger than the others. Note also that the nodes' intelligent and resilient increase their size, suggesting that they were more times named.

This procedure was extended to the whole data set, allowing us to identify the network constituent elements (i.e., stereotypical features) and the pattern of association between them (i.e., stereotypes structure). The network analysis was supported by Gephi 0.9.2 software (Bastian et al., 2009).

### 2.2. Results and discussion

The results for descriptive text analyses about gender stereotypes and metastereotypes features are depicted in the Supplementary material (Section S2). We conducted network analyses for each gender stereotype to identify how the features mentioned above were interconnected to form the structure of gender stereotypes. We calculated their communities—i.e., (sub)set of nodes whose connections are stronger than their connection with the rest of the nodes in the network (Blondel et al., 2008)—and visualized them by colors. Moreover, we visualize the networks according to the features of the nodes: the bigger nodes—i.e., those named more times together with other features—are in the central part of the network, and the more related nodes—i.e., those named more times together—are placed closer to each other.

#### 2.2.1. Female stereotype

**Figure 2:**
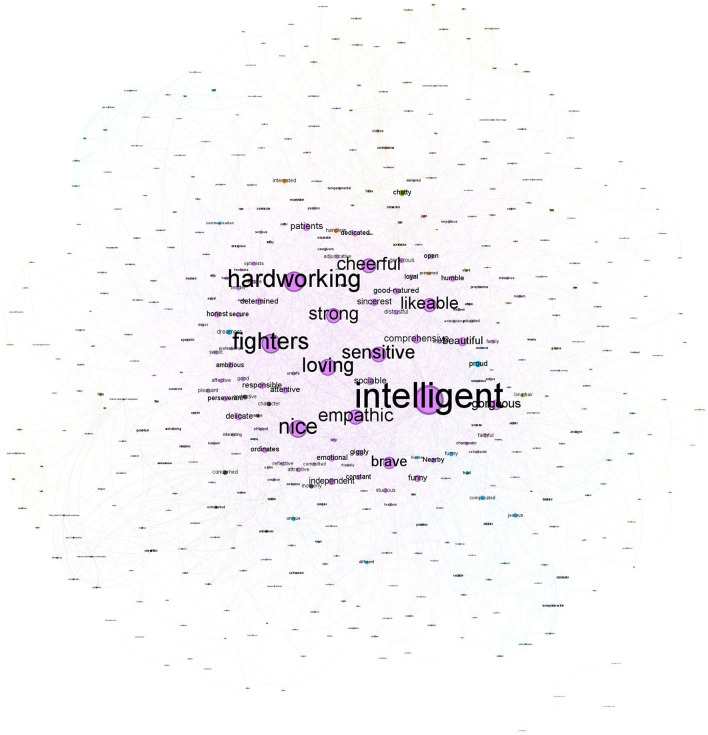
Network for female stereotypes mentioned by men.

The female stereotype that men showed was composed of a network that included 853 nodes, with 4,398 co-occurrences collapsed in 3,474 edges. Communality analyses showed three large clusters of features. The strongest co-occurrences were *intelligent*-*strong* (22), *loving–intelligent* (18), *intelligent–hardworking* (17), *fighter–hardworking* (17), *and fighter-intelligent* (15). As notably seen in Figure 2, the largest community (colored by light purple) represents 26% of the network, and their nodes with the highest centrality degree—i.e., the number of relationships that each node has with other nodes—are *intelligent* (215^2^), *hardworking* (149), and *fighters* (139). These results suggest that these features had more relevance in this community, given they were the most connected (Brandes et al., 2005). Nevertheless, there are additional communities, although with lower representation. The second community (colored by green) represents 7.5% of the network, and their nodes with the highest degree are *brave* (67), *patience* (35), and *proud* (34). Finally, the third community (colored by blue) represents 5.39% of the network, and their nodes with the highest degree are *chatty* (84), *long hair* (71), and *hips* (38).

**Figure 3:**
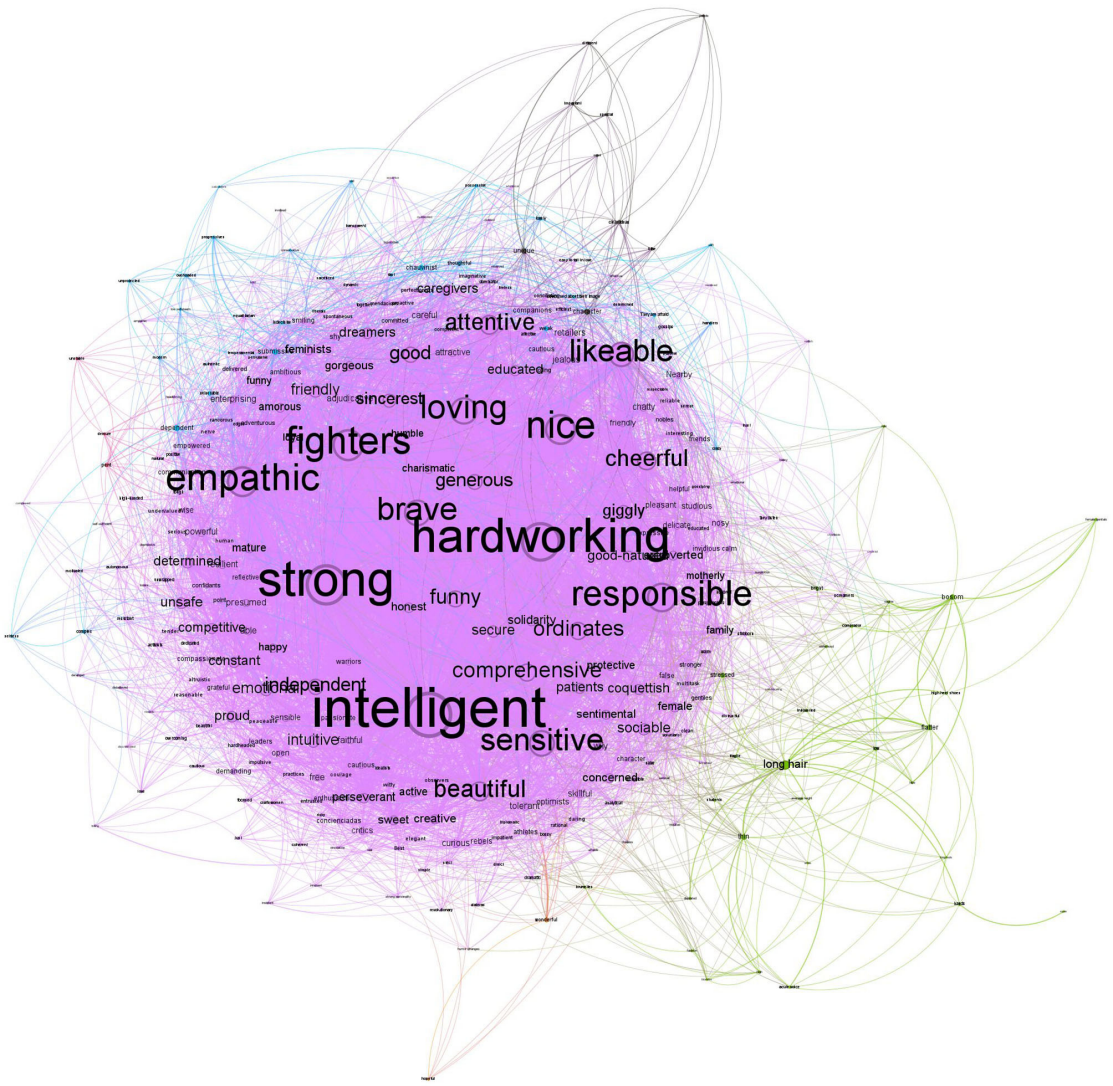
Network for female stereotypes mentioned by women.

The female stereotype that women provided (in-group stereotype) was composed of a network that included 853 nodes, with 13,600 co-occurrences collapsed in 7,711 edges. The strongest co-occurrences were *intelligent*-*strong* (92), *hardworking–intelligent* (86), *fighter–strong* (66), *strong–hardworking* (66), *and fighter-hardworking* (64). As seen in Figure 3, the most extensive community (colored by light purple) represents 49.36% of the network and their nodes with the highest degree are similar to those that men provided: *intelligent* (352), *strong* (312), and *hardworking* (299). Similarly to the female stereotype projected by men, there are additional communities but with lower representation. The second community (colored by green) represents 10.79% of the network, and their nodes with the highest degree are *long hair* (67), *thin* (35), and *bosom* (34). Finally, the third community (colored by blue) represents 8.68% of the network and their nodes with the highest degree are *unsafe* (84), *feminist* (71), and *dependent* (38).

In short, the female in-group stereotype was similar to the stereotypes that men have about women. These results suggest that the female stereotype (established by both women and men) is composed mainly by one community characterized by positive valued features linked among them such as *intelligent, strong, hardworking*, and *fighter*. The exception was that one of the main links in the women stereotypes projected by men was *intelligent*-*loving*, suggesting an important nuance in the meaning of the term intelligent when men attributed it to women. The weight this community has in the network suggests that the female stereotype is mainly, although not wholly, homogeneous. We should note that although most central features in this community might be considered agentic features, there are also other features more related to communion (e.g., sensitive, emphatic, and loving). The results also showed that there were additional small communities that characterized the female stereotype. Both women and men conveyed their female stereotype with physical features—i.e., *long hair, thin, bosom*, and *hips*.

Moreover, the female in-group stereotype also included a small community characterized by traditional features that reflect some type of vulnerability (e.g., *unsafe* and *dependent*). It is noteworthy that in this community, the feature of *feminist* also appeared. However, this feature should be interpreted into their network context, not only by their direct meaning. The label feminist has been traditionally stigmatized and used by some people as a negative feature to describe women (Anastosopoulos and Desmarais, 2015). Our results suggest that this is the connotation in which some (very few) women used this label. Indeed, what our network analyses suggest is that when women used *feminist* to describe the female stereotype, they also tended to use *unsafe, dependent, submissive*, or *pent*. Therefore, the meaning that they seemed to attribute to the feminist label could be linked to the stigmatized sense of the word. Yet, it is also likely that feminist is linked to undermining features as a way of compensating such vulnerability.

#### 2.2.2. Male stereotype

**Figure 4:**
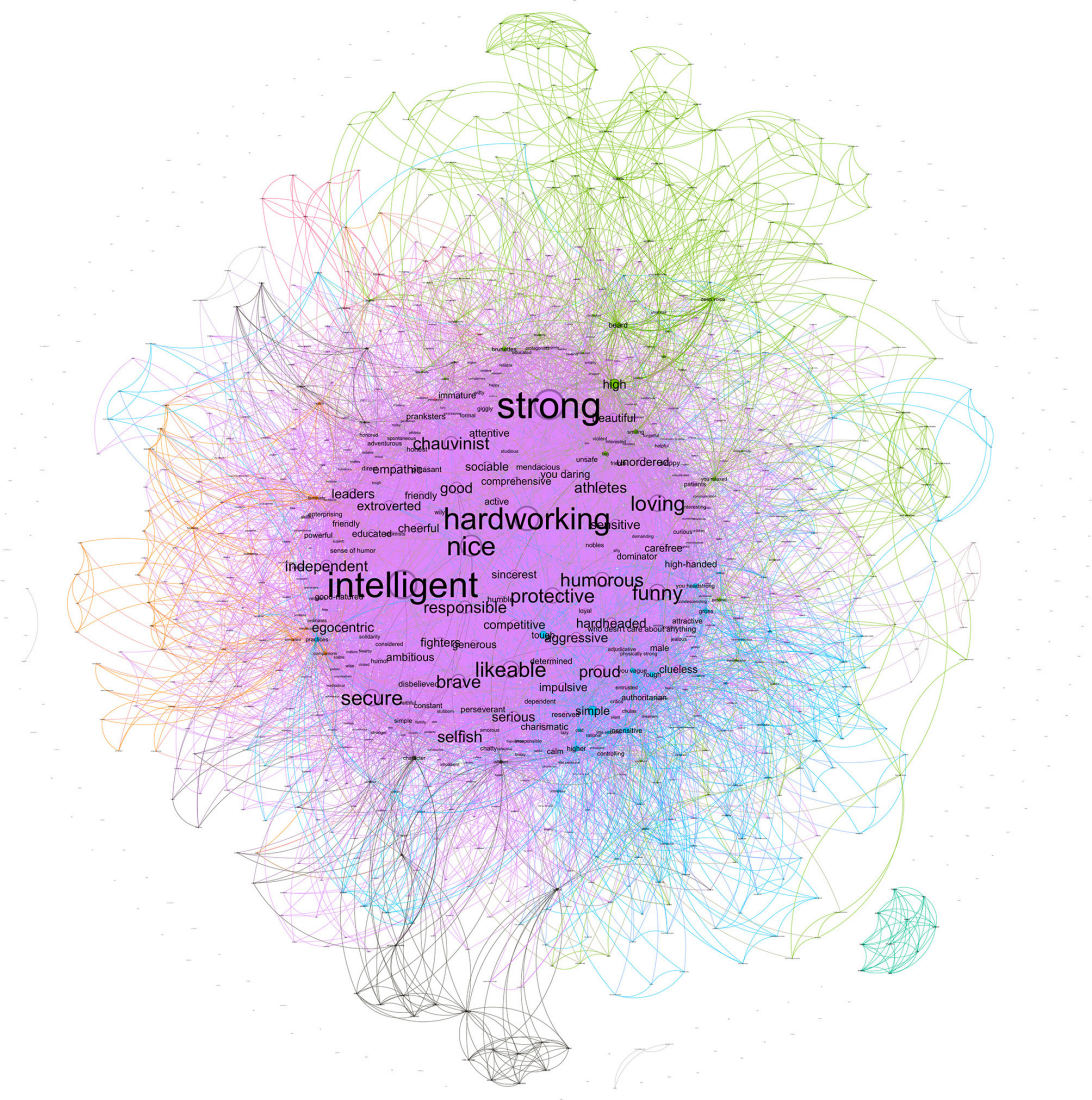
Network for male stereotypes mentioned by women.

The male stereotype provided by women included 995 nodes with 11,794 co-occurrences collapsed in 8,378 edges. The strongest co-occurrences were *intelligent*-*hardworking* (44), *strong–intelligent* (40), *strong–hardworking* (36), *nice–intelligent* (32), *and nice-hardworking* (29). As seen in Figure 4, the largest community (colored by light purple) represents 46.53% of the network, and their nodes with the highest degree are similar to those that women answered: *strong* (320), *intelligent* (305), and *hardworking* (259). There are additional communities but with lower representation. The second community (colored by green) represents 13.67% of the network, and their nodes with the highest degree are *high* (102), *beard* (58), and *brunettes* (45). Finally, the third community (colored by blue) represents 10.95% of the network, and their nodes with the highest degree are *simple* (96), *tough* (75), and *rough* (54).

**Figure 5:**
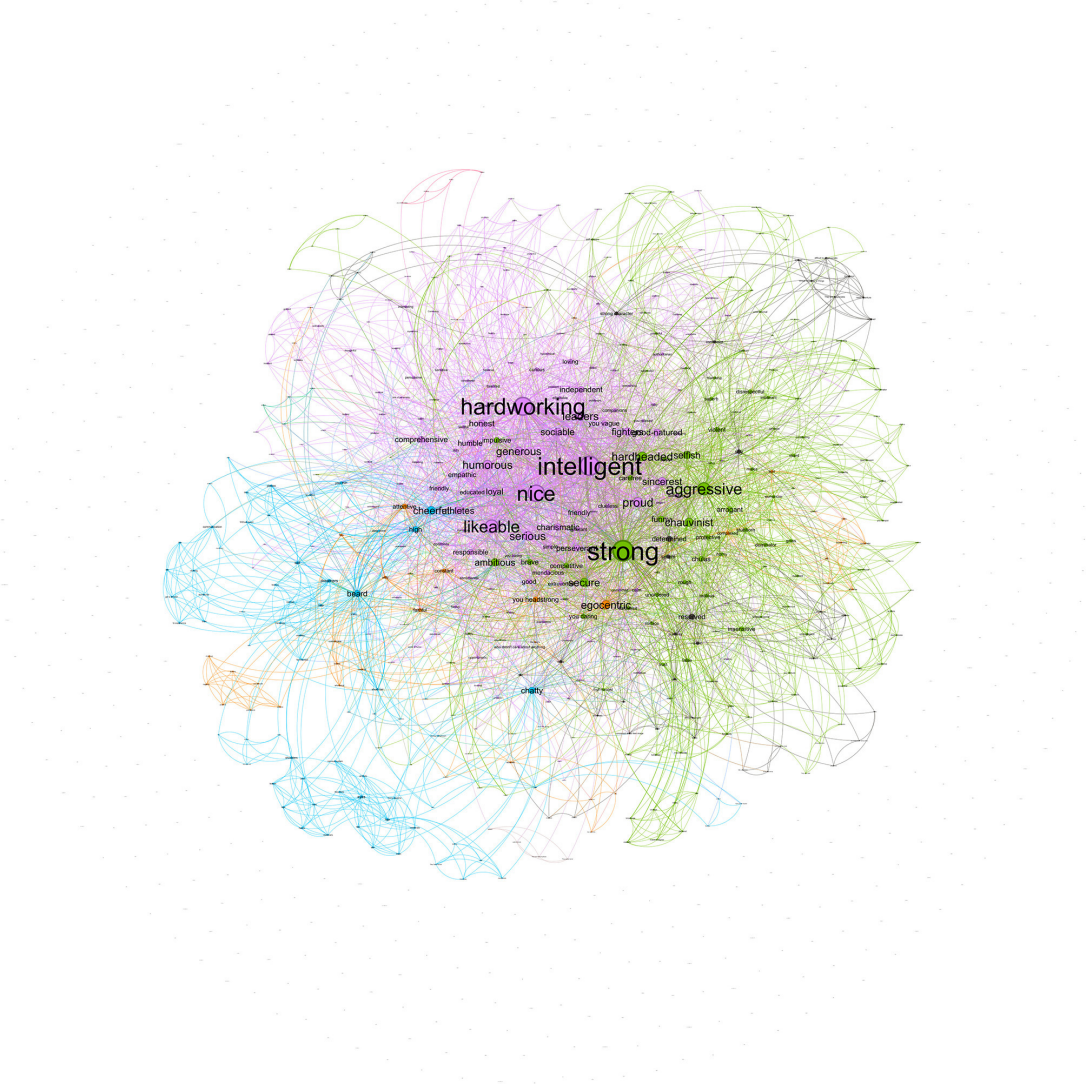
Network for male stereotypes mentioned by men.

The male in-group stereotype included 995 nodes, with 4,145 co-occurrences collapsed in 3,582 edges. The strongest co-occurrences were *intelligent*-*hardworking* (13), *strong–intelligent* (13), *likable–intelligent* (9), *nice–intelligent* (9), *and strong-hardworking* (9). As seen in Figure 5, the largest community (colored by light purple) represents 19.6% of the network, and their nodes with the highest degree are similar to those women answered about male stereotype: *intelligent* (141), *hardworking* (131), and *nice* (120). Interestingly, there is a second extensive community. This second community (colored by green) represents 13.67% of the network, and their nodes with the highest degree are *strong* (149), *aggressive* (89), and *hardheaded* (64), showing a relatively strong interconnection between those features: *strong-aggressive* (4), *strong-hardheaded* (3), and *strong-hardheaded* (2). Finally, the third community (colored by blue) represents 7.14% of the network, and their nodes with the highest degree are *cheerful* (56), *chatty* (45), and *beard* (44).

These results suggest that male stereotypes that both women and men answered were different. First, the male stereotype women referred to has a structure with a big cluster, whereas male in-group stereotype has a structure with two big clusters. Second, unlike female stereotypes in which we could distinguish between traditional and modern female stereotypes, the structure of male stereotype seems to reflect larger communities that could be considered as a traditional stereotype. Indeed, the male stereotype that women projected was represented mainly by one single community characterized by features such as *strong, intelligent*, and *hardworking*, which we could define as a traditional male stereotype (Prentice and Carranza, 2002). This community was similar to one of the two large communities identified in the male in-group stereotype (colored by light purple in Figure 5). The other larger community identity for the male in-group stereotype (colored by green in Figure 5) was characterized by the features of *strong, aggressive*, and *hardheaded*, which are also defined as traditional ones. Following previous research that evaluated the valence of male traditional stereotypes in the Spanish context (Martínez-Marín and Martínez, 2019), we can interpret that the main difference between both communities is their valence: Men characterized as intelligent and hardworking are positive features of traditional men, whereas men characterized as aggressive and hardheaded are negative features of traditional men. Especially the latter community suggests a widespread stereotype of hegemonic masculinity (Messerschmidt, 2019) characterized by toughness, among others (Levant et al., 2013).

It is worth noting the ambiguity of the valence of the feature *strong*. Indeed, one of the strengths of the network perspective is that we should interpret the meaning of a feature by taking into account its interactions with other features. When we evaluated the valence of strong in the sentiments analyses, it was considered a positive feature, in line with previous research (Martínez-Marín and Martínez, 2019; see Section S2 in Supplementary material). In this regard, women project a homogeneous male stereotype characterized by strong, together with intelligent and hardworking, which suggests it can be interpreted as a positive feature. Indeed, we considered this part of the stereotype as a positive traditional male stereotype. However, the male in-group stereotype used more frequently the word strong together with aggressive and hardheaded, which could be considered negative features. Certainly, the image of a strong man is different whether he is also described as intelligent and as hardworking or as aggressive and hardheaded.

Finally, a similarity between the male stereotype projected by both women and men, and shared with the female stereotype, is that a small part of the network is based on physical features (e.g., *high, beard, brunettes, beautiful*, and *athletes*).

#### 2.2.3. Female metastereotypes

**Figure 6:**
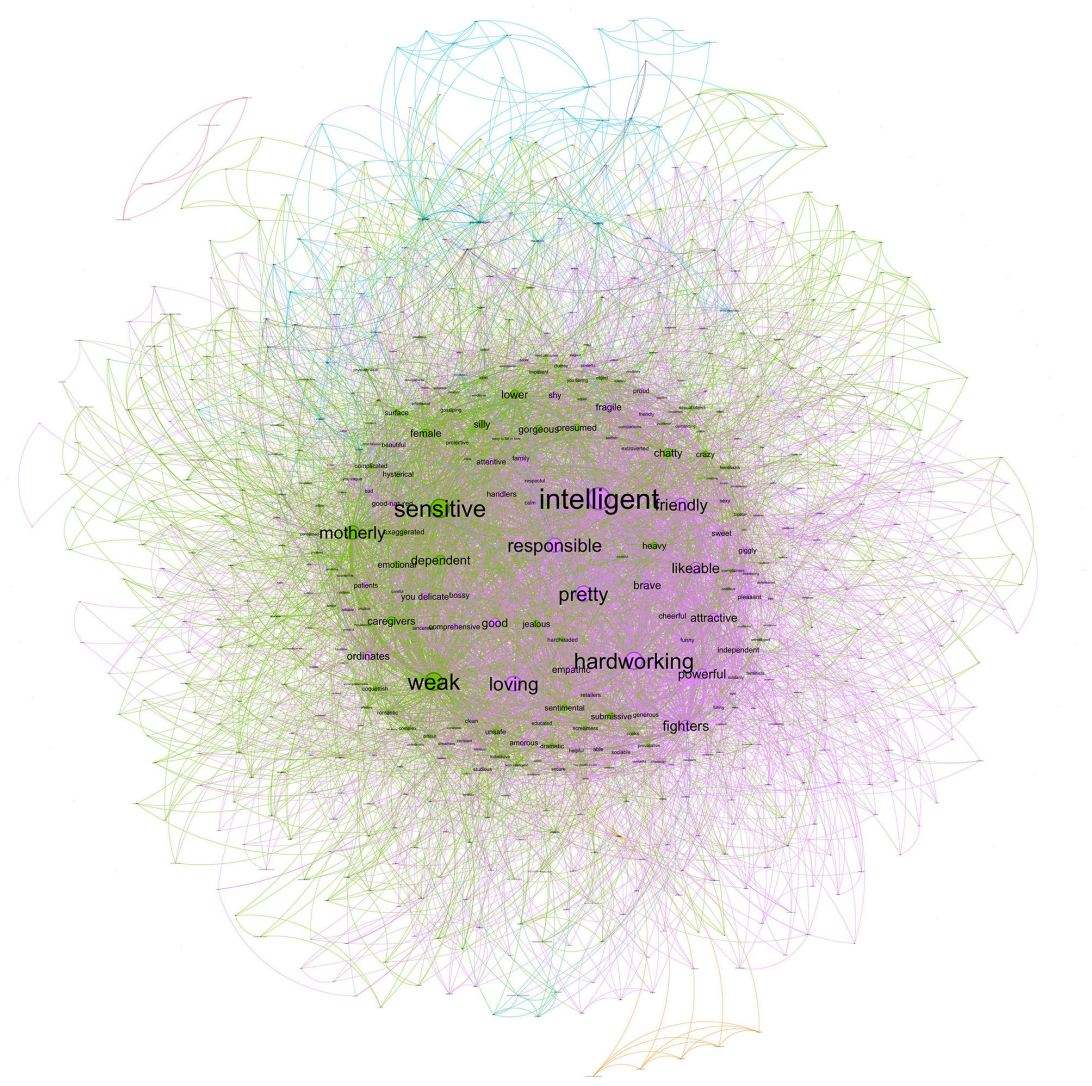
Network for female metastereotype.

The female metastereotype included 632 nodes, with 9,742 co-occurrences collapsed in 6,851 edges. The strongest co-occurrences were *intelligent*-*pretty* (29), *weak–sensitive* (28), *intelligent–hardworking* (27), *friendly–intelligent* (25), *and sensitive-intelligent* (24). As seen in Figure 6, the two largest communities (colored by light purple and green) are quite similar in size representing 45.41% and 44.94% of the network, respectively. The nodes with the highest degree of the first community are *sensitive* (238), *weak* (226), and *motherly* (172). The nodes with the highest degree of the second community are *intelligent* (288), *hardworking* (222), and *pretty* (200). Finally, the third community (colored by blue) represents 5.22% of the network, and their nodes with the highest degree are *made-up* (27), *long hair* (26), and *friendship* (22).

These results suggest that the structure is heterogeneous because it is represented mainly by two communities. One community seems to reflect the traditional female stereotype which is characterized by the features of *sensitive, weak*, and *motherly*. By contrast, the other community is characterized by the features of *intelligent* and *hardworking*, which seem to reflect the modern female stereotype. However, there is a crucial nuance in this last community given the role of *pretty*, which qualify importantly the meaning of *intelligent* and *hardworking*. It must be emphasized that these two communities are similar to women's in-group stereotypes except for their relative size. Whereas, women's in-group stereotype is mainly representative of modern female stereotypes, considering the traditional one is only a small part of the network; when women think of how men stereotype them, both traditional and modern communities take on a similar relevance. These results suggest that most women tend to in-group stereotype as modern women (i.e., *intelligent, hardworking*, and *strong*), although a few of them keep the traditional stereotype (i.e., *dependent* and *submissive)*. However, when looking at their beliefs about how men see them, women show more disagreement in the features mentioned. There is a similar number of women who think men see them as modern and traditional. Nevertheless, this does not appear to be the case. In other words, the female stereotype (both projected by men and women) is more homogeneous than the female metastereotype. Both women and men think about the female stereotype mainly as a modern stereotype. We did not find evidence that the female stereotypes projected by men reflected traditional features. Moreover, a part of the female metastereotype is represented by physical features—i.e., *long hair, thin, bosom, hips*—similar to the female stereotype.

#### 2.2.4. Male metastereotypes

**Figure 7:**
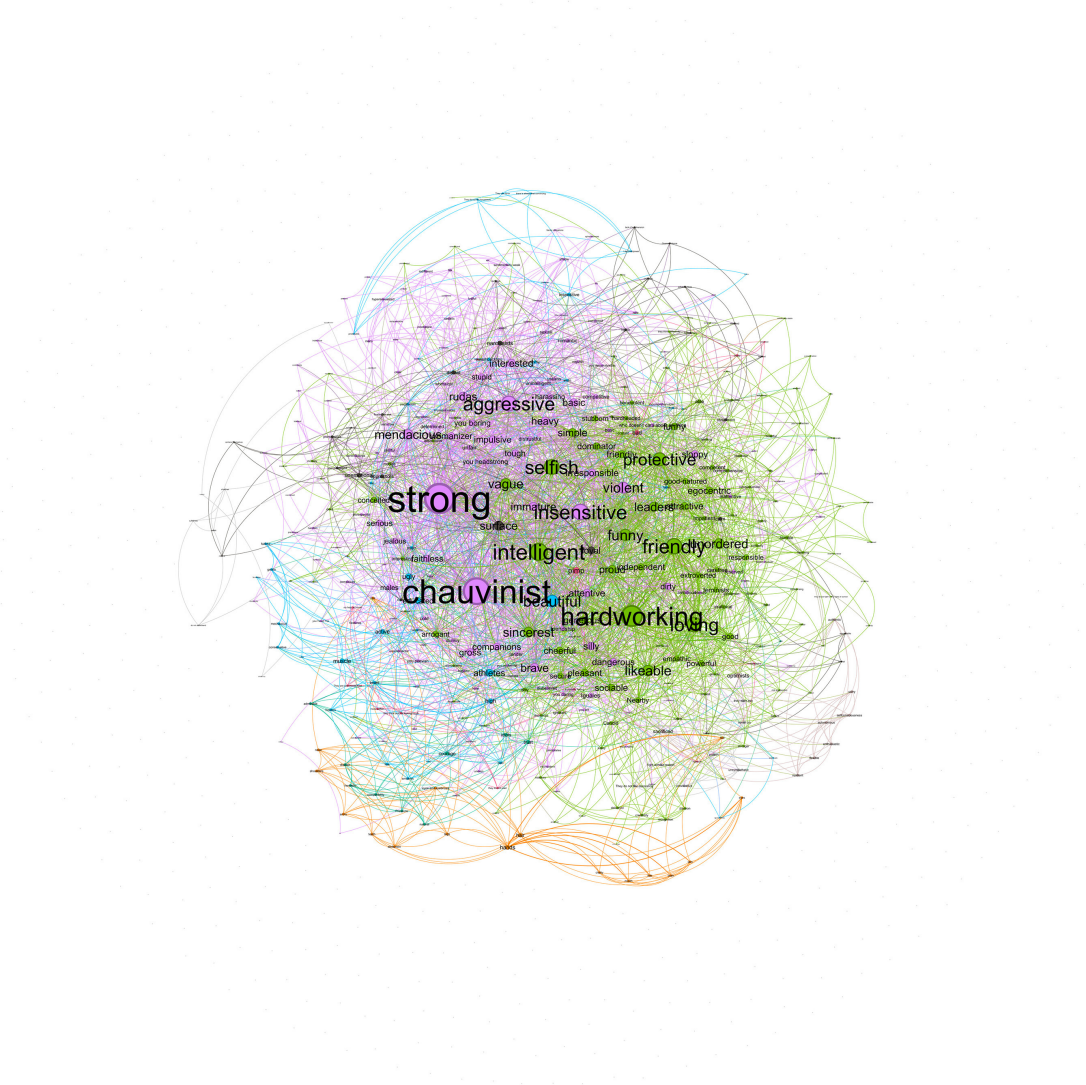
Network for male metastereotypes.

The male metastereotype included 412 nodes, with 3,115 co-occurrences collapsed in 2,756 edges. The strongest co-occurrences were *chauvinist*-*strong* (8), *strong–intelligent* (8), *strong–brave* (8), *strong–aggressive* (6), *and protective-hardworking* (5). As seen in Figure 7, the largest communities (colored by light purple) represent 32.04% of the network, and their nodes with the highest degree are *hardworking* (94), *intelligent* (78), and *friendly* (70). The second community (colored by green) represents 23.54% of the network, and their nodes with the highest degree are *strong* (135), *chauvinist* (120), and *insensitive* (71). There is a third community (colored by blue) which represents 13.83% of the network, and their nodes with the highest degree are *violent* (49), *heavy* (35), and *basic* (33). Finally, the fourth community (colored by dark gray) represents 9.71% of the network, and their nodes with the highest degree are *beautiful* (53), *athletes* (31), and *educated* (25).

The male metastereotype's structure was heterogeneous, given that it was mainly built from two main communities. These communities were quite similar to those found for the male in-group stereotype: traditional positive male stereotype (i.e., *intelligent* and *hardworking*) and traditional negative male stereotype (i.e., *strong, aggressive*, and *hardheaded*). Therefore, the male metastereotype was quite similar to the male in-group stereotype but different from the male stereotype women project, which was homogeneous with a main community of the traditional positive male stereotype (i.e., *intelligent* and *hardworking*). The main network analysis results are summarized in Supplementary material (Section S4). To see a summary of all the network communities, see Table 1.

**Table 1:** Summary of the female and male stereotypes that compose the network communities.

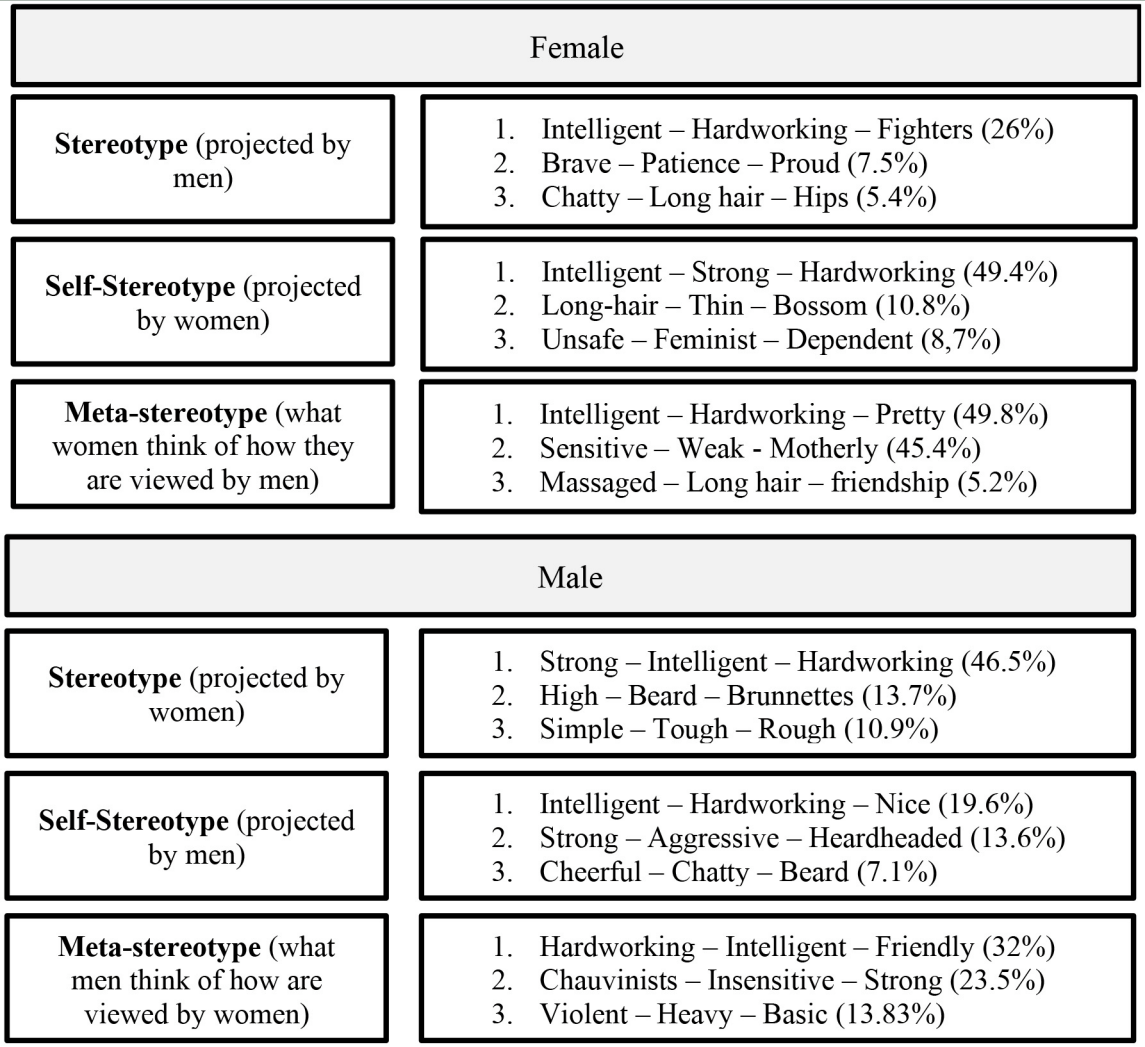

Three communities per each network were selected, and percentages indicate the number of nodes pertaining to that community.

## 3. General discussion

The aim of this research was to map the structure of gender stereotypes and metastereotypes as complex systems *via* a network approach. Our results suggest that the female stereotype women project is similar to that of how men project it. Both women and men have a female stereotype that is mainly homogeneous. The main community that characterizes women as intelligent, hardworking, strong, and fighters can be interpreted as a modern female stereotype. However, the male stereotype women project is different from how men project it. Women characterize men mainly homogeneously, with the main community using features such as intelligent, hardworking, strong, and fighters, which could be interpreted as a traditional positive male stereotype. However, men characterize themselves heterogeneously. There is a community similar to that of what women project (i.e., intelligent, hardworking, and fighter), and another community characterized by features such as strong, aggressive, and hardheaded, which could be interpreted as a traditional negative male stereotype.

Additionally, according to our results, women and men show a heterogeneous metastereotype. Two central communities characterize both female and male metastereotypes. The female metastereotype is characterized by what could be defined as a modern female stereotype as well as a traditional female stereotype. The male metastereotype, on the contrary, is characterized by what could be defined as a traditional positive male stereotype as well as a traditional negative male stereotype. We highlight that both women and men project a metastereotype differently than the gender stereotype that the opposite gender projects. Therefore, it seems that both women and men project a distorted metastereotype in comparison with the opposite gender views, but for a different reason. Women think that men's beliefs about women are different from the beliefs that women have about themselves, which seems not to be the case because both women and men seem to share a similar female stereotype. However, men think that women's beliefs about men are the same that men have about themselves, which seems not to be the case because women and men showed different male stereotypes. It is worth highlighting this point because of the mismatch between metastereotype and how out-group stereotype in-group might condition the intergroup relationships (Scherer et al., 2015).

As we mentioned above, the network approach provides us with the full interaction between nodes (Cramer et al., 2010)—i.e., features—which means that each feature attributed to each gender stereotype should be interpreted according to their interactions with other features. We already discussed above the feminist label applies to the female in-group stereotype and the strong valence applies to the male stereotype. In this regard, we must stress how the feature *strongly* interacted with other features in both female and male stereotypes. *Strong* is a feature that appeared in the male and female stereotypes with a high frequency and acted as an important node within the networks. However, in the female stereotype, *strong* had a relatively high co-occurrence with features such as *empathic, loving, sensitive*, and *likable*. By contrast, in men's in-group stereotype and metastereotype, *strong* had a relatively high co-occurrence with features such as *aggressive, chauvinist, insensitive*, and *hardheaded*. These results suggest that the notion that participants had when characterizing women as strong was different than when characterizing men. The fact that one feature can be interpreted with different nuances according to other features being presented before it is a well-known phenomenon from Asch's (1946) classic studies. In this line, our results provide evidence that a single feature might have different meanings when it is linked to different features.

### 3.1. Theoretical implications

The network approach allowed us to operationalize the mental image that our sample had about gender stereotypes recovering the traditional conception of stereotypes (Lippmann, 1922). Our results suggest that these mental images are not wholly homogeneous. Although some gender stereotypes are mainly, but not fully, homogeneous; there are always small proportions of the network that represent alternative views of gender stereotypes. These results might shed light on the literature that addresses whether current gender stereotypes have changed over time. Our results suggest that the female stereotype is mainly homogenous, represented by a mental image that women project with features attributed traditionally to men (e.g., intelligent, hardworking, and strong). This result is in line with research that showed women were seen as more competent than they were several decades ago (Duehr and Bono, 2006; Eagly et al., 2020). However, at the same time, a small proportion of women's in-group stereotype is represented by features typically attributed to traditional women (i.e., unsafe, dependent, submissive, or pent). This result is in line with the research that showed that the female stereotype had not changed over time (Haines et al., 2016). According to our results, to some extent, both images live together in society. Although, according to our results, modern female stereotypes seem more prevalent than traditional ones. How both modern and traditional female images coexist is particularly striking in the female metastereotype. Indeed, in the female metastereotype, both communities have similar weights in the network. These results suggest that for some women, there is a mismatch between how they in-group stereotype and how they think that men see them. Given the importance that the metastereotype has in the intergroup relationship (Gómez, 2002; Babbitt et al., 2018), it would be worthy for future research to explore how the interaction between the female in-group stereotype and metastereotype might affect the social interaction between group genders.

Moreover, the current study suggests that features attributed to the male stereotype are those traditionally attributed to them. These results are in line with research that has shown how the features attributed to men have not changed in the last decades (Donnelly and Twenge, 2017). Our results suggest that these traditional features might be differentiated by two mental images, which we define as traditional positive and negative male stereotypes. However, one of the advantages of our approach is that participants were able to show freely the features attributed to men, which were then organized according to their co-occurrence. This approach allows us to look closer at the connotations of each community identified. In this regard, it is worth focusing on the community that we have labeled as the traditional positive male stereotype. Although we have considered this community as traditional because the features with the highest degree are traditionally attributed to men, we also need to note the other features with high degrees in the community. Features such as nice, likable, generous, friendly, or loving might qualify the notion that this is a traditional community given that it includes communal features (Abele and Wojciszke, 2014; Wojciszke and Abele, 2019). Although including these features in this community seems to be the exception more than the norm, this result might point out that the male stereotype includes communal features in their mental image, which is in line with the literature about new masculinities (Kaplan et al., 2017).

Nonetheless, from a network perspective, the features form dynamic and complex systems because of the interactions among themselves (Cramer et al., 2010; Robinaugh et al., 2020). Therefore, we have shown that we should not interpret a single feature as isolated, but in interaction with those that tend to go together—i.e., strong with empathic or aggressive. Then, taking into account the full network of gender stereotypes' implications, it can be assumed that they are not interchangeable indicators that could be averaged on general indices. This is particularly important in some cases where features have contrary nuances. Identical features in different structures may cease to be identical, and they might even become opposed meanings. For instance, *sensitive* has a relatively high co-occurrence with *strong* in the female in-group stereotype appearing in the community that reflects modern female stereotypes. Nevertheless, in the female metastereotype, *sensitive* appeared more times together with *weak*, which is why it is included in the community of traditional female metastereotype. Therefore, the image projected when someone says that women are sensitive might be quite different if they are thinking sensitive and strong, rather than if they are thinking sensitive and weak. Future research should take these results into consideration, especially those that use close-ended questions to ask about features attributed to gender stereotypes and are averaged in a general index pre-set.

In this line, stereotypical features usually are considered linked with a specific valence—i.e., positive or negative (e.g., Martínez-Marín and Martínez, 2019). However, from a network approach, the features might not be perceived as positive or negative themselves. Otherwise, it might depend on what other features interact. For instance, when strong is together with intelligent and hardworking might be considered as more positive than when it is together with aggressive and hardheaded (see male stereotypes). Future research might explore how perceived valence might change according to the position of the features in the whole network.

The current research provided an alternative approach to study gender stereotypes. We propose to understand gender stereotypes as complex systems (Ladyman and Wiesner, 2020), which allow us to know their structure. Knowing their structure provided crucial information to evaluate whether they are homogeneous or heterogeneous, structural similarities between gender stereotypes provided for women and men, or between gender stereotypes and metastereotypes. Moreover, their structure shed light on how stereotypical gender features interact with each other qualifying their meanings. The network approach opens an avenue for future research in the study of stereotypes. Here, we focus on descriptive gender stereotypes, but future research might focus on prescriptive gender stereotypes. Moreover, future research might extend the network approach to the stereotype of other groups (e.g., Black-White; Republican-Democrat, high-low social class).

We conducted a procedure of co-occurrence to build the structure of gender stereotypes and focus on the communities or clusters of this structure. However, network analyses provided other parameters and other procedures to conduct them. For instance, centrality parameters provide information about the inter-connectedness of the features (Bringmann et al., 2019). Then, these parameters help to identify the important aspect of the network as central features which might be potential predictors of outcomes (Contreras et al., 2019). Moreover, it has been developed other procedures to conduct network analyses such as partial correlation network (Epskamp et al., 2018). Partial correlation network estimates the edges as correlations controlling for all other correlations in the network. This procedure allows, for instance, to compare quantitatively two networks. Centrality parameters and the chance of comparing networks might inspire future research questions in the study of stereotypes.

### 3.2. Practice implications

Identifying the structure of the current gender stereotypes is important for practice. Our results showed that there is a contradictory view of how each gender thinks the other gender sees them. Women are still thinking that men see them in traditional way as weak and sensitive people, and men think women see them as mainly competent but also tough. However, neither case corresponds to how people perceive other persons of the opposite gender. We are therefore dealing with a possible source of conflict between groups. People's misperception of how the other gender sees their gender can negatively impact—directly or indirectly—the behavior toward the out-groups (e.g., O'Brien et al., 2018). As such, showing the actual misperception of how we see others and others see us (stereotypes), how we see our gender group (in-group stereotypes), and how we think that *others* see *us (metastereotype)* will allow people to understand some of the underpinnings of their behavior and hopefully engage strategies to change it for the better.

Moreover, knowing the structure of gender stereotypes allows us to detect central features within the network stereotypes and to examine how those features are interconnected brings opportunities for social interventions. As posited by Asch (1946), the features in the stereotype formation can be central or peripheral according to their relevance within the network. Therefore, affecting one central feature is likely to change the overall structure of the whole stereotype. Previous research has shown that political identities are highly influential over the whole person's network of belief systems (Brandt, 2020) and that intervening specific symptoms of some mental diseases can accelerate the therapeutic treatments (Fried et al., 2020). Therefore, finding and altering central nodes of the network stereotypes could have practical implications to change gender stereotypes. In this sense, practitioners can develop social interventions aimed at challenging (boosting) central negative (positive) features that facilitate harmonious relationships between women and men.

Moreover, knowing the structure of gender stereotypes can provide a check about to what extent the policies implemented against gender inequality are proving to be effective and are permeating social representations of society. One of the strategies of these policies is to foster a change in gender roles, which should lead to a change in gender stereotypes (Eagly et al., 2020). The homogeneity of female stereotype and in-group stereotype as a competence-related cluster might be a consequence of the increasing participation of women in the labor force and higher education. Indeed, intelligence and hardworking are features required in the labor and educational field. Although there is still a long way to go in women's participation in gender-incongruent roles, such as STEM field, the structure of the current female stereotype suggests that the social changes in gender roles have permeated in female stereotypes. However, the changes in men's roles have been slower and late, which might explain why they are permeated less in male stereotypes. For instance, paternity leave in Spain has increased from 4 weeks in 2017 to 24 weeks in 2022. Our results point out that some features such as nice, generous, or loving, required to care to the children, are uncommon in the structure of male stereotype. Updating the structure of male stereotype in the close future might work as an indirect proof of the effectiveness of these policies.

### 3.3. Limitations and future research directions

Finally, there are also some limitations in this research that indicate key directions for further research. First, we used a convenient sample limited to a single country—i.e., Spain. Moreover, the political orientation of our sample was asymmetric toward the left wing, which might be conditioning our results given that previous research has shown that political orientation is related to sexist ideology (Hodson and MacInnis, 2017). Therefore, this procedure might constrain the generalizability of our findings (Simons et al., 2017). Further research with representative samples in Spain and in other countries might help to expand the scope of these results in other contexts for depicting the mental image of gender stereotypes.

Second, participants freely answered the features that they attributed to women and men. Although this procedure has some advantages as discussed above, it also has the drawback that it might trigger a social desirability bias. Moreover, procedures for making quantitative comparisons among networks are still under development, which leads us to ground our analyses on word frequencies and theoretical qualitative interpretations. Future research might use close-ended items to measure the features attributed to gender stereotypes and build psychometric networks that use partial correlations that allow for quantitative comparisons (Epskamp et al., 2018).

Third, text analytics have additional limitations to take into account. For instance, the lemmatization process helps to reduce large amounts of redundant terms and make them handy to manipulate and interpret, but this advantage has a cost since it is possible to lose sight of important nuances underlying similar words. We controlled this limitation by doing the lemmatization process by hand, allowing researchers to retain substantive information. However, this is a time-consuming task that quickly becomes unfeasible when much more information is available. Therefore, a combination of manual and automatic coding would be an optimal solution to overcome this limitation.

Fourth, this research was conducted in the Spanish language and translated into English. There are linguistic terms in the Spanish language with two or more potential translations (e.g., *trabajador*) and others without direct translation (e.g., *pacientes and chulas*). Therefore, the final terminology might not be a crystal-clear mirror of the original language used. Indeed, even using the same language, the features used for depicting gender stereotypes can be shaped by the cultural context and diverse connotations linked to similar words. Future research could conduct a similar procedure in other languages and cultural contexts to examine to what extent the structure of gender stereotypes is shared across different contexts.

Fifth, reviews of research that followed a network approach have suggested that networks can be unstable and difficult to replicate (Robinaugh et al., 2020), even though some networks have been replicated (Brandt, 2020). Moreover, we should note that our sample size of men was lower than that of women; therefore, the network structure of the men's answers provided may be more unstable because it has fewer components (Epskamp et al., 2018). Therefore, further research would be needed to increase the size of participants and responses to explore whether gender stereotype networks are stable across groups. The replication of network stereotypes, however, should be taken with caution because cultural, political, and historical events can exert substantive influence on how people perceive others. As such, any replication attempt should account for cultural differences that help to explain and understand the emerging pattern of gender stereotypes.

Sixth, there are additional potential explanations for the differences between stereotypes and metastereotypes found. For example, metastereotypes include motivational components (e.g., in-group favoritism) that might drive the differences found in their structures in comparison with stereotypes. Future studies should address how these motivational components could affect or be affected by the structure of metastereotypes and their implications (e.g., valence). Moreover, gender stereotypes and metastereotypes are broad categories that could be divided into subcategories (e.g., by sexual orientation, ethnicity, and social class). Previous research has found that there are differences between subgroups of stereotypes. For instance, DeWall et al. (2005) found that participants perceived six different subgroups of women: professional, feminist, homemaker, female athlete, beauty, and temptress. Therefore, the differences found could be due to that participants were thinking in different subcategories. In this line, there is a large literature about the intersectionality between gender and other stereotypes such as ethnicity, and socioeconomic status, which contain unique features that are not the result of adding gender stereotypes to other stereotypes (Ghavami and Peplau, 2013). For example, recent research has shown that women and men in poverty are perceived differently (Alcañiz-Colomer et al., 2023). More specifically, women are viewed as less personally responsible for being poor than men. This difference in perception of women and men has important implications given that this greater internal attribution of responsibility for being poor, in turn, was linked to less support for social protection policies when the recipients are men. Future research should, therefore, explore differences in the structure of subcategories in gender stereotypes and metastereotypes. Finally, we built our research from a binary perspective (female vs. male) as a general starting point. However, addressing gender as a more fluid construct rather than a categorical one could provide additional insights into the nature of gender stereotypes as recent research highlights the ongoing shift in gender conceptualization away from binary categories to a broader spectrum (Abed et al., 2019; Wickham et al., 2023).

In sum, our findings could be extended and refined in several ways. Thus, one of our contributions to this field is to apply text and network analyses to map gender stereotypes from a complex dynamic perspective in such a way that it can be easily followed by other researchers using open-source tools (see Supplementary material for code and files: https://osf.io/cmf6a/).

## 4. Conclusion

Gender stereotypes are key to understand gender inequalities. This is why knowing their structure provides useful information to examine to what extent they are heterogeneous or homogenous, which allows for checking their potential changes. Our results suggest that although the female stereotype is homogenous, the female metastereotype is more heterogeneous and coexists with both modern and traditional female stereotypes. Moreover, the male stereotype women project by participants is homogenous and characterized by a traditional positive male stereotype. By contrast, male in-group stereotypes and metastereotypes are heterogeneous, characterized by two communities: traditional positive male stereotypes and traditional negative male stereotypes. Moreover, stereotypical gender features seem to interact with each other to build gender stereotypes. Addressing gender stereotypes as a complex system from a network approach provides a fertile ground for future research.

## Data availability statement

The datasets presented in this study can be found in online repositories. The names of the repository/repositories and accession number(s) can be found below: Open Science Framework (https://osf.io/cmf6a/).

## Ethics statement

The studies involving human participants were reviewed and approved by Ethical Clearance ID: 1696/CEIH/2020. The patients/participants provided their written informed consent to participate in this study.

## Author contributions

ÁS-R and EM-B contributed to the conception and design of the study and organized the database. ÁS-R and EG-S performed the analyses of the data. ÁS-R wrote the first draft of the manuscript. EM-B and EG-S wrote sections of the manuscript. All the authors contributed to manuscript revisions, read, and approved the submitted version.

## Footnotes

^1^Participants who answered “Other” or did not answer the gender question were not included in the analyses of metastereotypes.

^2^Values within the parentheses indicate the node degree of centrality, which is interpreted as the number of connections of this feature with other features.
