# Supplementary material for: Mapping gender stereotypes: a network analysis approach
Source: Front Psychol. 2023 Jul 18;14:1193866. doi: 10.3389/fpsyg.2023.1193866 (PMC10393260; doi:10.3389/fpsyg.2023.1193866)
Supplement: Supplementary file 1 [file Data_Sheet_1.docx]

Supplementary Material

Mapping Gender Stereotypes: A Network Analysis Approach

Ángel Sánchez-Rodríguez^1^, Eva Moreno-Bella^2*^, Efraín García-Sánchez^3^

*** Correspondence:** Eva Moreno-Bella: [embella@psi.uned.es](mailto:embella@psi.uned.es)

**Note**. In this file, we present Characteristic of the sample (Section S1), frequency and sentiment analysis (Section S2), details of network analyses (Section S3), and general features of the gender (meta)stereotypes networks (Section S4).

**Section S1**

**Characteristics of the Sample**

| **Table S1**  *Characteristics of the Sample* | | | |
| --- | --- | --- | --- |
| Socioeconomic status by household income | Category | Frequency | Percentage |
|  | Less than 650€ | 26 | 4.28 |
|  | Between 651–1.300€ | 150 | 24.67 |
|  | Between 1.301–1.950€ | 147 | 24.18 |
|  | Between 1.951–2.600€ | 108 | 17.76 |
|  | Between 2.601–3.250€ | 78 | 12.83 |
|  | Between 3.251–3.900€ | 42 | 6.91 |
|  | Between 3.901–4.550€ | 34 | 5.59 |
|  | Between 4.551–5.200€ | 8 | 1.32 |
|  | Between 5.201–5.800€ | 9 | 1.48 |
|  | More than 5.800€ | 6 | 0.99 |
|  | No information | 142 |  |
| Socioeconomic status by subjective social class | 1 | *0* | *0* |
|  | 2 | 12 | 1.95 |
|  | 3 | 44 | 7.15 |
|  | 4 | 108 | 17.56 |
|  | 5 | 120 | 19.51 |
|  | 6 | 174 | 28.29 |
|  | 7 | 115 | 18.7 |
|  | 8 | 38 | 6.18 |
|  | 9 | 2 | 0.33 |
|  | 10 | 2 | 0.33 |
|  | No information | 135 |  |
| Political ideology | Extremely left | 55 | 9.08 |
|  | Moderatly left | 256 | 42.24 |
|  | Slightly left | 117 | 19.31 |
|  | Center | 107 | 17.66 |
|  | Slighty right | 45 | 7.43 |
|  | Moderatly right | 20 | 3.30 |
|  | Extremely right | 6 | 0.99 |
|  | No information | 144 | NA |

**Section S2**

**Additional Analyses: Frequency and Sentiment Analysis.**

We conducted a descriptive frequency analysis to look at the prevalence of the attributes and applied a sentiment analysis to explore the differences in positive and negative features attributed to gender stereotypes per the participant’s gender.

### *Descriptive Textual Analysis for Gender (Meta)Stereotypes*

Participants provided 14,212 responses in total (*N_Stereo.Female_* = 5,259; *N_Stereo.Male_* = 4,891; *N_Meta-Stereo.Female_* = 3,033; *N_Meta-Stereo.Male_ =* 1,029). The frequency of answers in each gender (meta)stereotype can be found in Table S2. First, we did not find significant differences in the numbers of words related to male stereotype between women (*M* = 6.83, *SD* = 2.25) and men (*M* = 6.69, *SD* = 2.35), *t*(700) = -0.70, *p* = .487. However, women provided more words in the female stereotype (*M* = 7.34, *SD* = 2.23) than men (*M* = 6.92, *SD* = 2.30), *t*(700) = -2.22, *p* = .027. Finally there were no significant differences in the metastereotype between women (*M* = 5.87, *SD* = 3.01) and men (*M* = 5.44, *SD* = 2.95), *t*(700) = , *p* =.094. Across the three stereotypes, women tended to provide more words on average than men, although only in women stereotypes the differences were significant. This result might be an effect of the link between women traditional characteristics and linguistic skills and humanities disciplines (e.g., Hand et al., 2017).

Then, we present a frequency analysis of the words women and men used to characterises gender stereotypes. As shown in Table S3, the most frequent word used to describe the female (meta)stereotype was *intelligent.* This result suggests that competence features characterise both female stereotypes and metastereotypes. However, focusing on the next more frequently mentioned features, this feature seems to have a different connotation. In the case of the female stereotype, this word was followed by *hard hardworking,* *strong*, and *friendly*. However, in the case of female metastereotypes, the next words more frequently used words were *beautiful*, *weak*, and *sensitive*.

Regarding male (meta)stereotypes, the most frequent word used was *strong*. Nevertheless, the next most frequent words suggested an ambivalent mental image. On the one hand, people mentioned features as *friendly*, *intelligent*, and *hardworking*. However, male metastereotypes also were linked to features such as *chauvinist*.

| **Table S2.**  *Frequency of Answers in Gender Stereotypes and Meta-Stereotypes* | | | | | | |  |
| --- | --- | --- | --- | --- | --- | --- | --- |
| Number of words answered | Stereotypes | | |  | Meta-Stereotypes | | |
|  | Female | Male | |  | Female | Male | |
| 0 | 19 |  | 31 |  | 66 | 27 | |
| 1 | 3 |  | 4 |  | 2 | 1 | |
| 2 | 4 |  | 3 |  | 3 | 3 | |
| 3 | 8 |  | 8 |  | 9 | 4 | |
| 4 | 7 |  | 15 |  | 1 | 2 | |
| 5 | 207 |  | 274 |  | 159 | 72 | |
| 6 | 136 |  | 127 |  | 82 | 25 | |
| 7 | 72 |  | 51 |  | 50 | 16 | |
| 8 | 44 |  | 23 |  | 24 | 6 | |
| 9 | 9 |  | 5 |  | 13 | 3 | |
| 10 | 241 |  | 209 |  | 103 | 31 | |

| **Table S3.**  *Word Frequency of Gender Stereotypes and Meta-stereotypes* | | | | | | | | | | | | | | | | | | | | | | |
| --- | --- | --- | --- | --- | --- | --- | --- | --- | --- | --- | --- | --- | --- | --- | --- | --- | --- | --- | --- | --- | --- | --- |
| Female | | | | | | | | | | |  | Male | | | | | | | | | | |
| Stereotype  (projected by men) | | |  | Self-stereotype  (female stereotype projected  by women) | | |  | Meta-stereotype  (how women think they  are viewed by men | | |  | Stereotype  (projected by women) | | |  | Self-stereotype  (male stereotype projected  by men) | | |  | Meta-stereotype  (how men think they  are viewed by women | | |
| Word | Freq. | % |  | Word | Freq. | % |  | Word | Freq. | % |  | Word | Freq. | % |  | Word | Freq. | % |  | Word | Freq. | % |
| intelligent | 77 | 5.88 |  | intelligent | 216 | 5.75 |  | intelligent | 131 | 4.32 |  | strong | 140 | 4.01 |  | friendly | 45 | 3.74 |  | strong | 43 | 3.56 |
| hardworking | 49 | 3.74 |  | hardworking | 174 | 4.63 |  | beautiful | 110 | 3.63 |  | intelligent | 133 | 3.81 |  | strong | 42 | 3.49 |  | chauvinist | 31 | 2.56 |
| friendly | 44 | 3.36 |  | strong | 169 | 4.50 |  | weak | 103 | 3.40 |  | friendly | 133 | 3.81 |  | intelligent | 42 | 3.49 |  | friendly | 25 | 2.07 |
| strong | 35 | 2.67 |  | friendly | 145 | 3.86 |  | sensitive | 98 | 3.23 |  | hardworking | 124 | 3.55 |  | hardworking | 41 | 3.41 |  | hardworking | 23 | 1.90 |
| fighters | 34 | 2.60 |  | fighters | 123 | 3.27 |  | hardworking | 87 | 2.87 |  | jolly | 108 | 3.09 |  | jolly | 24 | 1.99 |  | intelligent | 21 | 1.74 |
| loving | 33 | 2.52 |  | loving | 98 | 2.61 |  | loving | 82 | 2.70 |  | likeable | 65 | 1.86 |  | stubborn | 20 | 1.66 |  | jolly | 15 | 1.24 |
| cheerful | 31 | 2.37 |  | empathic | 95 | 2.53 |  | friendly | 76 | 2.51 |  | loving | 61 | 1.75 |  | likeable | 19 | 1.58 |  | insensitive | 15 | 1.24 |
| beautiful | 27 | 2.06 |  | brave | 88 | 2.34 |  | likeable | 50 | 1.65 |  | secure | 55 | 1.57 |  | aggressive | 15 | 1.25 |  | loving | 14 | 1.16 |
| empathic | 27 | 2.06 |  | sensitive | 79 | 2.10 |  | responsible | 50 | 1.65 |  | protective | 48 | 1.37 |  | athletes | 14 | 1.16 |  | protective | 14 | 1.16 |
| sensitive | 26 | 1.99 |  | likeable | 75 | 2.00 |  | Motherly | 47 | 1.55 |  | brave | 46 | 1.32 |  | cheerful | 13 | 1.08 |  | aggressive | 13 | 1.08 |
| likeable | 24 | 1.83 |  | responsible | 73 | 1.94 |  | fighters | 46 | 1.52 |  | stubborn | 41 | 1.17 |  | sociable | 13 | 1.08 |  | selfish | 13 | 1.08 |
| brave | 22 | 1.68 |  | cheerful | 70 | 1.86 |  | strong | 43 | 1.42 |  | responsible | 39 | 1.12 |  | leaders | 13 | 1.08 |  | rude | 13 | 1.08 |
| determined | 15 | 1.15 |  | beautiful | 62 | 1.65 |  | attractive | 35 | 1.15 |  | chauvinist | 37 | 1.06 |  | secure | 12 | 1.00 |  | beautiful | 12 | 0.99 |
| jolly | 14 | 1.07 |  | jolly | 58 | 1.54 |  | dependent | 33 | 1.09 |  | cheerful | 36 | 1.03 |  | proud | 11 | 0.91 |  | violent | 11 | 0.91 |
| responsible | 11 | 0.84 |  | attentive | 45 | 1.20 |  | good | 32 | 1.06 |  | independent | 35 | 1.00 |  | fighters | 11 | 0.91 |  | likeable | 10 | 0.83 |
| comprehensive | 11 | 0.84 |  | comprehensive | 43 | 1.14 |  | brave | 30 | 0.99 |  | proud | 34 | 0.97 |  | egocentric | 11 | 0.91 |  | lazy | 10 | 0.83 |
| independent | 11 | 0.84 |  | independent | 39 | 1.04 |  | cheerful | 30 | 0.99 |  | simple | 34 | 0.97 |  | serious | 11 | 0.91 |  | brave | 10 | 0.83 |
| sincere | 11 | 0.84 |  | tidy | 36 | 0.96 |  | chatty | 30 | 0.99 |  | athletes | 33 | 0.94 |  | generous | 11 | 0.91 |  | leaders | 9 | 0.74 |
| attentive | 10 | 0.76 |  | good | 35 | 0.93 |  | tidy | 28 | 0.92 |  | selfish | 32 | 0.92 |  | honest | 11 | 0.91 |  | simple | 9 | 0.74 |
| emotional | 10 | 0.76 |  | generous | 34 | 0.90 |  | female | 27 | 0.89 |  | fighters | 29 | 0.83 |  | sincere | 10 | 0.83 |  | stubborn | 8 | 0.66 |
| *Note*. Percentages are calculated based on the total of words mention in each category (N_Stereo.Female_ = 1303; N_self-stero.Female_ = 3759; N_Meta-Stereo.Female_ = 3033; N_Stereo.Male_ = 3493; N_self-stero.Male_ = 1204; N_Meta-Stereo.Male_ = 1029). Complete data is available in the online supplementary material: https://osf.io/cmf6a/?view_only=467b4f4d02094cd8805a0fcd7569ac16 | | | | | | | | | | | | | | | | | | | | | | |

### *Sentiment Analysis for Gender Stereotypes*

Because gender stereotypes imply an evaluation of social groups (i.e., women and men), they also inevitably imply positive or negative connotations (Suitner & Maass, 2008); therefore, we decided to conduct a sentiment analysis to explore the word valence used to describe gender (meta)stereotypes. We conducted this analysis by using the sentiment dictionary Bing, implemented in the Tidytext set of packages for R software (Silge & Robinson, 2016). Table S3 depicts the most frequent words associated with each stereotype and metastereotype by the word valence. As shown in Table S3, positive words were more frequently used to describe female and male stereotypes and metastereotypes. These results suggest that gender (meta)stereotypes are mainly seen as positive. The main exception to this is the word *weak*, which women used to describe the female metastereotype. It is worth pointing out that this word is hardly used to describe female stereotypes.

We conducted a Pearson chi-square test to examine whether the valence of the word was related to the participant’s gender for each gender stereotype and metastereotype. Results showed that there was a statistically significant association between the participant’s gender and word sentiment. We found that men (vs. women) were less likely to use positive words when they referred to female stereotypes, χ^2^(1, 702) = 8.66, *p* = .003, OR = .70, 95% CI = [.55, .90]. Similarly, male participants were also less likely to use positive words when talking about male stereotypes compared to women: χ^2^(1, 702) = 12.26, *p* < .001, OR = .72, 95% CI = [.60, .87]. We also found that men (vs. women) were less likely to use positive words when talking about male metastereotypes, χ^2^(1, 702) = 29.16, *p* < .001, OR = .60, 95% CI = [.50, .73] (see Table S4 and S5).

These results suggest that women tend to use more positive features to describe any of the (meta)stereotypes than men do. Some researchers did not find the effect of the participants’ gender when measuring trait words and valence (e.g., Abele & Brückmuller, 2011), but others claimed the need to be cautious with this possibility (Oliveira et al., 2020). It worth noting that research about features attributed to people and valence usually selected previously the same number of positive and negative features and asked participants to rate them (e.g., Bruckmüller & Abele, 2013). By contrast, our bottom-up strategy allowed us to test the tendency of women and men to provide positive or negative features in characterizing the gender (meta)stereotype. A possible explanation of the responses’ pattern that we found could be that women used more positive words than men because they tend to be more oriented to maintaining good relationships as well as being kind and gentle with others. That is, as traditionally claimed, women tend to be more communal than men (Ellemers, 2018).

| **Table S4.**  *Contingency Table between Gender of Participant and Word Sentiment by Gender Stereotypes* | | | | |
| --- | --- | --- | --- | --- |
| Gender stereotypes | Gender | Word sentiment | |  |
|  |  | Negative | Positive | Total |
| Female | Female | 251 | 1958 | 2209 |
|  | Male | 114 | 622 | 736 |
|  | Total | 365 | 2580 |  |
|  |  |  |  |  |
| Male | Female | 541 | 1520 | 2061 |
|  | Male | 238 | 482 | 720 |
|  | Total | 779 | 2002 |  |

| **Table S5.**  *Word Frequency of Gender Stereotypes and Meta-stereotypes by Word Valence (Positive or Negative)* | | | | | | | | | | | |
| --- | --- | --- | --- | --- | --- | --- | --- | --- | --- | --- | --- |
|  | Female | | | | |  | Male | | | | |
|  | Positive | |  | Negative | |  | Positive | |  | Negative | |
|  | Word | Freq. |  | Word | Freq. |  | Word | Freq. |  | Word | Freq. |
| Stereotype |  |  |  |  |  |  |  |  |  |  |  |
|  | intelligent | 77 |  | concerned | 5 |  | strong | 140 |  | stubborn | 41 |
|  | friendly | 44 |  | distrustful | 5 |  | friendly | 133 |  | selfish | 32 |
|  | strong | 35 |  | complicated | 4 |  | intelligent | 133 |  | egocentric | 28 |
|  | loving | 33 |  | critics | 4 |  | jolly | 108 |  | aggressive | 23 |
|  | cheerful | 31 |  | insecure | 4 |  | loving | 61 |  | impulsive | 19 |
|  | beautiful | 27 |  | jealous | 4 |  | secure | 55 |  | arrogant | 18 |
|  | sensitive | 26 |  | manipulative | 4 |  | protective | 48 |  | conceited | 18 |
|  | brave | 22 |  | boastful | 3 |  | brave | 46 |  | messy | 18 |
|  | jolly | 14 |  | fragile | 3 |  | cheerful | 36 |  | clueless | 16 |
|  | comprehensive | 11 |  | impulsive | 3 |  | proud | 34 |  | apathetic | 12 |
| Self-stereotype |  |  |  |  |  |  |  |  |  |  |  |
|  | intelligent | 216 |  | insecure | 17 |  | friendly | 45 |  | stubborn | 20 |
|  | strong | 169 |  | flirty | 15 |  | intelligent | 42 |  | aggressive | 15 |
|  | friendly | 145 |  | concerned | 14 |  | strong | 42 |  | egocentric | 11 |
|  | loving | 98 |  | boastful | 11 |  | jolly | 24 |  | lazy | 10 |
|  | brave | 88 |  | stubborn | 10 |  | cheerful | 13 |  | arrogant | 9 |
|  | sensitive | 79 |  | submissive | 9 |  | sociable | 13 |  | selfish | 9 |
|  | cheerful | 70 |  | critics | 8 |  | secure | 12 |  | rude | 8 |
|  | beautiful | 62 |  | envious | 8 |  | generous | 11 |  | cocky | 7 |
|  | jolly | 58 |  | jealous | 7 |  | honest | 11 |  | impulsive | 7 |
|  | attentive | 45 |  | false | 6 |  | proud | 11 |  | violent | 5 |
| Meta-stereotype |  |  |  |  |  |  |  |  |  |  |  |
|  | intelligent | 131 |  | weak | 103 |  | strong | 43 |  | insensitive | 15 |
|  | beautiful | 110 |  | fragile | 23 |  | friendly | 25 |  | aggressive | 13 |
|  | sensitive | 98 |  | silly | 23 |  | intelligent | 21 |  | rude | 13 |
|  | loving | 82 |  | boastful | 20 |  | jolly | 15 |  | selfish | 13 |
|  | friendly | 76 |  | submissive | 20 |  | loving | 14 |  | violent | 11 |
|  | strong | 43 |  | manipulative | 19 |  | protective | 14 |  | lazy | 10 |
|  | attractive | 35 |  | jealous | 18 |  | beautiful | 12 |  | conceited | 8 |
|  | good | 32 |  | crazy | 17 |  | brave | 10 |  | immature | 8 |
|  | brave | 30 |  | stubborn | 16 |  | cheerful | 8 |  | liar | 8 |
|  | cheerful | 30 |  | hysterical | 15 |  | sincere | 8 |  | messy | 8 |
| *Note.* We selected the 10 most-frequent words linked to each gender (meta)stereotype by word valence. Full information is disclosed in the online supplementary material. | | | | | | | | | | | |

**Section S3**

**Details of network analyses.**

We first focused on (meta)stereotypes of women and then on (meta)stereotypes of men. Given that one of our main aims was to identify whether (meta)stereotypes have a single representation or if they are composed of several representations, in addition to the general structure of the network, we examined the network’s underlying structure by identifying the communities that compounded it. A community is a (sub)set of nodes which connections are stronger than their connection with the rest of the nodes in the network (Blondel et al., 2008; Radicchi et al., 2004). To do the analyses of the communities, we ran the Leiden clustering algorithm to identify the commonalities (Traag et al., 2019). We used the quality function of Modularity using a resolution of 0.7 with 1,000 iterations. This algorithm identified a large number of communities; however, we only considered those big enough to be established as a not residual answer (> 5%). To visualize the network, we used the Fruchterman and Reingold (1991) layout, which is a force-directed algorithm that displays the nodes in relation to their position to other nodes. This algorithm places the bigger nodes—i.e., those more times named—in the central part of the network. Moreover, the more related nodes—i.e., those named more times together—are placed closer to each other. Moreover, in the networks, we visualize the communities by colors. We should note that there is an imbalance with the number of participants by gender. Although this gender imbalance may affect the networks composition and the emergence of more communities, our substantive results (given our sample size) do not provide evidence of a clear gender bias in the formation of more heterogeneous networks. The whole list of nodes and their network features can be found in OSF (<https://osf.io/cmf6a/>).

**Section S4**

**General features of the gender (meta)stereotypes networks**

| Table S6.*General features of the gender (meta)stereotypes networks* | | | | | | | | |
| --- | --- | --- | --- | --- | --- | --- | --- | --- |
|  | Stereotype | | | | |  | Meta-Stereotypes | |
|  | Female | |  | Male | |  |  |  |
| Anwered by | Female | Male |  | Female | Male |  | Female | Male |
| Diameter | 6 | 5 |  | 5 | 4 |  | 4 | 5 |
| Average path length | 2.4 | 2.5 |  | 2.5 | 2.65 |  | 2.4 | 2.6 |
| Density | 0.02 | 0.01 |  | 0.02 | 0.01 |  | 0.03 | 0.03 |
| Degree average | 18.5 | 8.1 |  | 16.8 | 7.2 |  | 21.7 | 13.4 |
| Modularity quality | 0.34 | 0.42 |  | 0.37 | 0.49 |  | 0.34 | 0.50 |
| Number of communities identified | 195 | 411 |  | 203 | 512 |  | 8 | 12 |

**References**

Abele, A. E., & Bruckmüller, S. (2011). The bigger one of the “Big Two”? Preferential processing of communal information. *Journal of Experimental Social Psychology*, *47*(5), 935–948. <https://doi.org/10.1016/j.jesp.2011.03.028>

Bastian, M., Heymann, S., & Jacomy, M. (2009). Gephi: An open source software for exploring and manipulating networks. In *Third International ICWSM Conference* (pp. 361-362). <https://www.aaai.org/ocs/index.php/ICWSM/09/paper/view/154>

Blondel, V. D., Guillaume, J-L., Lambiotte, R., & Lefebvre, E. (2008). Fast unfolding of communities in large networks. *Journal of Statistical Mechanics: Theory and Experiment,* *10*, P10008. <https://doi.org/10.1088/1742-5468/2008/10/P10008>.

Bruckmüller, S., & Abele, A. (2013). The density of the big two: How are agency and communion structurally represented? *Social Psychology*, *44*(2), 63–74. <https://doi.org/10.1027/1864-9335/a000145>

Fruchterman, T. M. J., & Reingold, E. M. (1991). Graph drawing by force‐directed placement. *Software: Practice and Experience, 21*(11), 1129–1164. <https://doi.org/10.1002/spe.4380211102>

Hand, S., Rice, L., & Greenlee, E. (2017). Exploring teachers’ and students’ gender role bias and students’ confidence in STEM fields. *Social Psychology of Education,* *20***,**929–945 2017. <https://doi.org/10.1007/s11218-017-9408-8>

Oliveira, M., Garcia-Marques, T., Garcia-Marques, L., & Dotsch, R. (2020). Good to bad or bad to bad? What is the relationship between valence and the trait content of the Big Two? *European Journal of Social Psychology*, *50*(2), 463–483. <https://doi.org/10.1002/ejsp.2618>

Radicchi, F., Castellano, C., Cecconi, F., Loreto, V. & Parisi, D. (2004). Defining and identifying communities in networks. *Proceedings of the National Academy of Sciences, 101* (9), 2658-63. <https://doi.org/10.1073/pnas.0400054101>.

Silge, J., & Robinson, R. (2016). Tidytext: Text mining and analysis using tidy data principles in R. *The Journal of Open Source Software, 1*(3), 37. <https://doi.org/10.21105/joss.00037>

Suitner, C. & Maass, A. (2008). The role of valence in the perception of agency and communion. *European Journal of Social Psychology, 38*(7), 1073-1082. <https://doi.org/10.1002/ejsp.525>

Traag, V. A., Waltman, L., & van Eck, J. (2019). From Louvain to Leiden: Guaranteeing well-connected communities. *Nature, 2*, 1–12. <https://doi.org/10.1038/s41598-019-41695-z>
